# The language faculty that wasn't: a usage-based account of natural language recursion

**DOI:** 10.3389/fpsyg.2015.01182

**Published:** 2015-08-27

**Authors:** Morten H. Christiansen, Nick Chater

**Affiliations:** ^1^Department of Psychology, Cornell UniversityIthaca, NY, USA; ^2^Department of Language and Communication, University of Southern DenmarkOdense, Denmark; ^3^Haskins LaboratoriesNew Haven, CT, USA; ^4^Behavioural Science Group, Warwick Business School, University of WarwickCoventry, UK

**Keywords:** recursion, language evolution, cultural evolution, usage-based processing, language faculty, domain-general processes, sequence learning

## Abstract

In the generative tradition, the language faculty has been shrinking—perhaps to include only the mechanism of recursion. This paper argues that even this view of the language faculty is too expansive. We first argue that a language faculty is difficult to reconcile with evolutionary considerations. We then focus on recursion as a detailed case study, arguing that our ability to process recursive structure does not rely on recursion as a property of the grammar, but instead emerges gradually by piggybacking on domain-general sequence learning abilities. Evidence from genetics, comparative work on non-human primates, and cognitive neuroscience suggests that humans have evolved complex sequence learning skills, which were subsequently pressed into service to accommodate language. Constraints on sequence learning therefore have played an important role in shaping the cultural evolution of linguistic structure, including our limited abilities for processing recursive structure. Finally, we re-evaluate some of the key considerations that have often been taken to require the postulation of a language faculty.

## Introduction

Over recent decades, the language faculty has been getting smaller. In its heyday, it was presumed to encode a detailed “universal grammar,” sufficiently complex that the process of language acquisition could be thought of as analogous to processes of genetically controlled growth (e.g., of a lung, or chicken's wing) and thus that language acquisition should not properly be viewed as a matter of learning at all. Of course, the child has to home in on the language being spoken in its linguistic environment, but this was seen as a matter of setting a finite set of discrete parameters to the correct values for the target language—but the putative bauplan governing all human languages was viewed as innately specified. Within the generative tradition, the advent of minimalism (Chomsky, 1995) led to a severe theoretical retrenchment. Apparently baroque innately specified complexities of language, such as those captured in the previous Principles and Parameters framework (Chomsky, 1981), were seen as emerging from more fundamental language-specific constraints. Quite what these constraints are has not been entirely clear, but an influential article (Hauser et al., 2002) raised the possibility that the language faculty, strictly defined (i.e., not emerging from general-purpose cognitive mechanisms or constraints) might be very small indeed, comprising, perhaps, just the mechanism of recursion (see also, Chomsky, 2010). Here, we follow this line of thinking to its natural conclusion, and argue that the language faculty is, quite literally, empty: that natural language emerges from general cognitive constraints, and that there is no innately specified special-purpose cognitive machinery devoted to language (though there may have been some adaptations for speech; e.g., Lieberman, 1984).

The structure of this paper is as follows. In *The Evolutionary Implausibility of an Innate Language Faculty*, we question whether an innate linguistic endowment could have arisen through biological evolution. In *Sequence Learning ad the Basis for Recursive Structure*, we then focus on what is, perhaps, the last bastion for defenders of the language faculty: natural language recursion. We argue that our limited ability to deal with recursive structure in natural language is an acquired skill, relying on non-linguistic abilities for sequence learning. Finally, in *Language without a Language Faculty*, we use these considerations as a basis for reconsidering some influential lines of argument for an innate language faculty^1^.

## The evolutionary implausibility of an innate language faculty

Advocates of a rich, innate language faculty have often pointed to analogies between language and vision (e.g., Fodor, 1983; Pinker and Bloom, 1990; Pinker, 1994). Both appear to pose highly specific processing challenges, which seem distinct from those involved in more general learning, reasoning, and decision making processes. There is strong evidence that the brain has innately specified neural hardwiring for visual processing; so, perhaps we should expect similar dedicated machinery for language processing.

Yet on closer analysis, the parallel with vision seems to lead to a very different conclusion. The structure of the visual world (e.g., in terms of its natural statistics, e.g., Field, 1987; and the ecological structure generated by the physical properties of the world and the principles of optics, e.g., Gibson, 1979; Richards, 1988) has been fairly stable over the tens of millions of years over which the visual system has developed in the primate lineage. Thus, the forces of biological evolution have been able to apply a steady pressure to develop highly specialized visual processing machinery, over a very long time period. But any parallel process of adaptation to the linguistic environment would have operated on a timescale shorter by two orders of magnitude: language is typically assumed to have arisen in the last 100,000–200,000 years (e.g., Bickerton, 2003). Moreover, while the visual environment is stable, the linguistic environment is anything but stable. Indeed, during historical time, language change is consistently observed to be extremely rapid—indeed, the entire Indo-European language group may have a common root just 10,000 years ago (Gray and Atkinson, 2003).

Yet this implies that the linguistic environment is a fast-changing “moving target” for biological adaptation, in contrast to the stability of the visual environment. Can biological evolution occur under these conditions? One possibility is that there might be co-evolution between language and the genetically-specified language faculty (e.g., Pinker and Bloom, 1990). But computer simulations have shown that co-evolution between slowly changing “language genes” and more a rapidly change language environment does not occur. Instead, the language rapidly adapts, through cultural evolution, to the existing “pool” of language genes (Chater et al., 2009). More generally, in gene-culture interactions, fast-changing culture rapidly adapts to the slower-changing genes and not vice versa (Baronchelli et al., 2013a).

It might be objected that not all aspects of the linguistic environment may be unstable—indeed, advocates of an innate language faculty frequently advocate the existence of strong regularities that they take to be universal across human languages (Chomsky, 1980; though see Evans and Levinson, 2009). Such universal features of human language would, perhaps, be stable features of the linguistic environment, and hence provide a possible basis for biological adaptation. But this proposal involves a circularity—because one of the reasons to postulate an innate language faculty is to explain putative language universals: thus, such universals cannot be assumed to pre-exist, and hence to provide a stable environment for, the evolution of the language faculty (Christiansen and Chater, 2008).

Yet perhaps a putative language faculty need not be a product of biological adaptation at all—could it perhaps have arisen through exaptation (Gould and Vrba, 1982): that is, as a side-effect of other biological mechanisms, which have themselves adapted to entirely different functions (e.g., Gould, 1993)? That a rich innate language faculty (e.g., one embodying the complexity of a theory such as Principles and Parameters) might arise as a distinct and autonomous mechanism by, in essence, pure chance seems remote (Christiansen and Chater, 2008). Without the selective pressures driving adaptation, it is highly implausible that new and autonomous piece of cognitive machinery (which, in traditional formulations, the language faculty is typically assumed to be, e.g., Chomsky, 1980; Fodor, 1983) might arise from the chance recombination of pre-existing cognitive components (Dediu and Christiansen, in press).

These arguments do not necessarily count against a very minimal notion of the language faculty, however. As we have noted, Hauser et al. (2002) speculate that the language faculty may consist of nothing more than a mechanism for recursion. Such a simple (though potentially far-reaching) mechanism could, perhaps, have arisen as a consequence of a modest genetic mutation (Chomsky, 2010). We shall argue, though, that even this minimal conception of the contents of the language faculty is too expansive. Instead, the recursive character of aspects of natural language need not be explained by the operation of a dedicated recursive processing mechanism at all, but, rather, as emerging from domain-general sequence learning abilities.

## Sequence learning as the basis for recursive structure

Although recursion has always figured in discussions of the evolution of language (e.g., Premack, 1985; Chomsky, 1988; Pinker and Bloom, 1990; Corballis, 1992; Christiansen, 1994), the new millennium saw a resurgence of interest in the topic following the publication of Hauser et al. (2002), controversially claiming that recursion may be the only aspect of the language faculty unique to humans. The subsequent outpouring of writings has covered a wide range of topics, from criticisms of the Hauser et al. claim (e.g., Pinker and Jackendoff, 2005; Parker, 2006) and how to characterize recursion appropriately (e.g., Tomalin, 2011; Lobina, 2014), to its potential presence (e.g., Gentner et al., 2006) or absence in animals (e.g., Corballis, 2007), and its purported universality in human language (e.g., Everett, 2005; Evans and Levinson, 2009; Mithun, 2010) and cognition (e.g., Corballis, 2011; Vicari and Adenzato, 2014). Our focus here, however, is to advocate a usage-based perspective on the processing of recursive structure, suggesting that it relies on evolutionarily older abilities for dealing with temporally presented sequential input.

### Recursion in natural language: What needs to be explained?

The starting point for our approach to recursion in natural language is that what needs to be explained is the observable human ability to process *recursive structure*, and not recursion as a hypothesized part of some grammar formalism. In this context, it is useful to distinguish between two types of recursive structures: tail recursive structures (such as 1) and complex recursive structures (such as 2).

(1) The mouse bit the cat that chased the dog that ran away.
(2) The dog that the cat that the mouse bit chased ran away.

Both sentences in (1) and (2) express roughly the same semantic content. However, whereas the two levels of tail recursive structure in (1) do not cause much difficulty for comprehension, the comparable sentence in (2) with two center-embeddings cannot be readily understood. Indeed, there is a substantial literature showing that English doubly center-embedded sentences (such as 2) are read with the same intonation as a list of random words (Miller, 1962), cannot easily be memorized (Miller and Isard, 1964; Foss and Cairns, 1970), are difficult to paraphrase (Hakes and Foss, 1970; Larkin and Burns, 1977) and comprehend (Wang, 1970; Hamilton and Deese, 1971; Blaubergs and Braine, 1974; Hakes et al., 1976), and are judged to be ungrammatical (Marks, 1968). Even when facilitating the processing of center-embeddings by adding semantic biases or providing training, only little improvement is seen in performance (Stolz, 1967; Powell and Peters, 1973; Blaubergs and Braine, 1974). Importantly, the limitations on processing center-embeddings are not confined to English. Similar patterns have been found in a variety of languages, ranging from French (Peterfalvi and Locatelli, 1971), German (Bach et al., 1986), and Spanish (Hoover, 1992) to Hebrew (Schlesinger, 1975), Japanese (Uehara and Bradley, 1996), and Korean (Hagstrom and Rhee, 1997). Indeed, corpus analyses of Danish, English, Finnish, French, German, Latin, and Swedish (Karlsson, 2007) indicate that doubly center-embedded sentences are almost entirely absent from spoken language.

By making complex recursion a built-in property of grammar, the proponents of such linguistic representations are faced with a fundamental problem: the grammars generate sentences that can never be understood and that would never be produced. The standard solution is to propose a distinction between an infinite linguistic *competence* and a limited observable psycholinguistic *performance* (e.g., Chomsky, 1965). The latter is limited by memory limitations, attention span, lack of concentration, and other processing constraints, whereas the former is construed to be essentially infinite in virtue of the recursive nature of grammar. There are a number of methodological and theoretical issues with the competence/performance distinction (e.g., Reich, 1969; Pylyshyn, 1973; Christiansen, 1992; Petersson, 2005; see also Christiansen and Chater, Forthcoming 2016). Here, however, we focus on a substantial challenge to the standard solution, deriving from the considerable variation across languages and individuals in the use of recursive structures—differences that cannot readily be ascribed to performance factors.

In a recent review of the pervasive differences that can be observed throughout all levels of linguistic representations across the world's current 6–8000 languages, Evans and Levinson (2009) observe that recursion is not a feature of every language. Using examples from Central Alaskan Yup'ik Eskimo, Khalkha Mongolian, and Mohawk, Mithun (2010) further notes that recursive structures are far from uniform across languages, nor are they static within individual languages. Hawkins (1994) observed substantial offline differences in perceived processing difficulty of the same type of recursive constructions across English, German, Japanese, and Persian. Moreover, a self-paced reading study involving center-embedded sentences found differential processing difficulties in Spanish and English (even when morphological cues were removed in Spanish; Hoover, 1992). We see these cross-linguistic patterns as suggesting that recursive constructions form part of a linguistic system: the processing difficulty associated with specific recursive constructions (and whether they are present at all) will be determined by the overall distributional structure of the language (including pragmatic and semantic considerations).

Considerable variations in recursive abilities have also been observed developmentally. Dickinson (1987) showed that recursive language production abilities emerge gradually, in a piecemeal fashion. On the comprehension side, training improves comprehension of singly embedded relative clause constructions both in 3–4-year old children (Roth, 1984) and adults (Wells et al., 2009), independent of other cognitive factors. Level of education further correlates with the ability to comprehend complex recursive sentences (Dąbrowska, 1997). More generally, these developmental differences are likely to reflect individual variations in experience with language (see Christiansen and Chater, Forthcoming 2016), differences that may further be amplified by variations in the structural and distributional characteristics of the language being spoken.

Together, these individual, developmental and cross-linguistic differences in dealing with recursive linguistic structure cannot easily be explained in terms of a fundamental recursive competence, constrained by fixed biological constraints on performance. That is, the variation in recursive abilities across individuals, development, and languages are hard to explain in terms of performance factors, such as language-independent constraints on memory, processing or attention, imposing limitations on an otherwise infinite recursive grammar. Invoking such limitations would require different biological constraints on working memory, processing, or attention for speakers of different languages, which seems highly unlikely. To resolve these issues, we need to separate claims about *recursive mechanisms* from claims about *recursive structure*: the ability to deal with a limited amount of recursive structure in language does not necessitate the postulation of recursive mechanisms to process them. Thus, instead of treating recursion as an *a priori* property of the language faculty, we need to provide a mechanistic account able to accommodate the actual degree of recursive structure found across both natural languages and natural language users: no more and no less.

We favor an account of the processing of recursive structure that builds on construction grammar and usage-based approaches to language. The essential idea is that the ability to process recursive structure does not depend on a built-in property of a competence grammar but, rather, is an acquired skill, learned through experience with specific instances of recursive constructions and limited generalizations over these (Christiansen and MacDonald, 2009). Performance limitations emerge naturally through interactions between linguistic experience and cognitive constraints on learning and processing, ensuring that recursive abilities degrade in line with human performance across languages and individuals. We show how our usage-based account of recursion can accommodate human data on the most complex recursive structures that have been found in naturally occurring language: center-embeddings and cross-dependencies. Moreover, we suggest that the human ability to process recursive structures may have evolved on top of our broader abilities for complex sequence learning. Hence, we argue that language processing, implemented by domain-general mechanisms—not recursive grammars—is what endows language with its hallmark productivity, allowing it to “*…make infinite employment of finite means,”* as the celebrated German linguist, Wilhelm von Humboldt (1836/1999: p. 91), noted more than a century and a half ago.

### Comparative, genetic, and neural connections between sequence learning and language

Language processing involves extracting regularities from highly complex sequentially organized input, suggesting a connection between general sequence learning (e.g., planning, motor control, etc., Lashley, 1951) and language: both involve the extraction and further processing of discrete elements occurring in temporal sequences (see also e.g., Greenfield, 1991; Conway and Christiansen, 2001; Bybee, 2002; de Vries et al., 2011, for similar perspectives). Indeed, there is comparative, genetic, and neural evidence suggesting that humans may have evolved specific abilities for dealing with complex sequences. Experiments with non-human primates have shown that they can learn both fixed sequences, akin to a phone number (e.g., Heimbauer et al., 2012), and probabilistic sequences, similar to “statistical learning” in human studies (e.g., Heimbauer et al., 2010, under review; Wilson et al., 2013). However, regarding complex recursive non-linguistic sequences, non-human primates appear to have significant limitations relative to human children (e.g., in recursively sequencing actions to nest cups within one another; Greenfield et al., 1972; Johnson-Pynn et al., 1999). Although more carefully controlled comparisons between the sequence learning abilities of human and non-primates are needed (see Conway and Christiansen, 2001, for a review), the currently available data suggest that humans may have evolved a superior ability to deal with sequences involving complex recursive structures.

The current knowledge regarding the *FOXP2* gene is consistent with the suggestion of a human adaptation for sequence learning (for a review, see Fisher and Scharff, 2009). *FOXP2* is highly conserved across species but two amino acid changes have occurred after the split between humans and chimps, and these became fixed in the human population about 200,000 years ago (Enard et al., 2002). In humans, mutations to *FOXP2* result in severe speech and orofacial motor impairments (Lai et al., 2001; MacDermot et al., 2005). Studies of *FOXP2* expression in mice and imaging studies of an extended family pedigree with *FOXP2* mutations have provided evidence that this gene is important to neural development and function, including of the cortico-striatal system (Lai et al., 2003). When a humanized version of *Foxp2* was inserted into mice, it was found to specifically affect cortico-basal ganglia circuits (including the striatum), increasing dendrite length and synaptic plasticity (Reimers-Kipping et al., 2011). Indeed, synaptic plasticity in these circuits appears to be key to learning action sequences (Jin and Costa, 2010); and, importantly, the cortico-basal ganglia system has been shown to be important for sequence (and other types of procedural) learning (Packard and Knowlton, 2002). Crucially, preliminary findings from a mother and daughter pair with a translocation involving *FOXP2* indicate that they have problems with both language and sequence learning (Tomblin et al., 2004). Finally, we note that sequencing deficits also appear to be associated with specific language impairment (SLI) more generally (e.g., Tomblin et al., 2007; Lum et al., 2012; Hsu et al., 2014; see Lum et al., 2014, for a review).

Hence, both comparative and genetic evidence suggests that humans have evolved complex sequence learning abilities, which, in turn, appear to have been pressed into service to support the emergence of our linguistic skills. This evolutionary scenario would predict that language and sequence learning should have considerable overlap in terms of their neural bases. This prediction is substantiated by a growing bulk of research in the cognitive neurosciences, highlighting the close relationship between sequence learning and language (see Ullman, 2004; Conway and Pisoni, 2008, for reviews). For example, violations of learned sequences elicit the same characteristic event-related potential (ERP) brainwave response as ungrammatical sentences, and with the same topographical scalp distribution (Christiansen et al., 2012). Similar ERP results have been observed for musical sequences (Patel et al., 1998). Additional evidence for a common domain-general neural substrate for sequence learning and language comes from functional imaging (fMRI) studies showing that sequence violations activate Broca's area (Lieberman et al., 2004; Petersson et al., 2004, 2012; Forkstam et al., 2006), a region in the left inferior frontal gyrus forming a key part of the cortico-basal ganglia network involved in language. Results from a magnetoencephalography (MEG) experiment further suggest that Broca's area plays a crucial role in the processing of musical sequences (Maess et al., 2001).

If language is subserved by the same neural mechanisms as used for sequence processing, then we would expect a breakdown of syntactic processing to be associated with impaired sequencing abilities. Christiansen et al. (2010b) tested this prediction in a population of agrammatic aphasics, who have severe problems with natural language syntax in both comprehension and production due to lesions involving Broca's area (e.g., Goodglass and Kaplan, 1983; Goodglass, 1993—see Novick et al., 2005; Martin, 2006, for reviews). They confirmed that agrammatism was associated with a deficit in sequence learning in the absence of other cognitive impairments. Similar impairments to the processing of musical sequences by the same population were observed in a study by Patel et al. (2008). Moreover, success in sequence learning is predicted by white matter density in Broca's area, as revealed by diffusion tensor magnetic resonance imaging (Flöel et al., 2009). Importantly, applying transcranial direct current stimulation (de Vries et al., 2010) or repetitive transcranial magnetic stimulation (Uddén et al., 2008) to Broca's area during sequence learning or testing improves performance. Together, these cognitive neuroscience studies point to considerable overlap in the neural mechanisms involved in language and sequence learning^2^, as predicted by our evolutionary account (see also Wilkins and Wakefield, 1995; Christiansen et al., 2002; Hoen et al., 2003; Ullman, 2004; Conway and Pisoni, 2008, for similar perspectives).

### Cultural evolution of recursive structures based on sequence learning

Comparative and genetic evidence is consistent with the hypothesis that humans have evolved more complex sequence learning mechanisms, whose neural substrates subsequently were recruited for language. But how might recursive structure recruit such complex sequence learning abilities? Reali and Christiansen (2009) explored this question using simple recurrent networks (SRNs; Elman, 1990). The SRN is a type of connectionist model that implements a domain-general learner with sensitivity to complex sequential structure in the input. This model is trained to predict the next element in a sequence and learns in a self-supervised manner to correct any violations of its own expectations regarding what should come next. The SRN model has been successfully applied to the modeling of both sequence learning (e.g., Servan-Schreiber et al., 1991; Botvinick and Plaut, 2004) and language processing (e.g., Elman, 1993), including multiple-cue integration in speech segmentation (Christiansen et al., 1998) and syntax acquisition (Christiansen et al., 2010a). To model the difference in sequence learning skills between humans and non-human primates, Reali and Christiansen first “evolved” a group of networks to improve their performance on a sequence-learning task in which they had to predict the next digit in a five-digit sequence generated by randomizing the order of the digits, 1–5 (based on a human task developed by Lee, 1997). At each generation, the best performing network was selected, and its initial weights (prior to any training)—i.e., their “genome”—was slightly altered to produce a new generation of networks. After 500 generations of this simulated “biological” evolution, the resulting networks performed significantly better than the first generation SRNs.

Reali and Christiansen (2009) then introduced language into the simulations. Each miniature language was generated by a context-free grammar derived from the grammar skeleton in Table 1. This grammar skeleton incorporated substantial flexibility in word order insofar as the material on the right-hand side of each rule could be ordered as it is (right-branching), in the reverse order (left-branching), or have a flexible order (i.e., the constituent order is as is half of time, and the reverse the other half of the time). Using this grammar skeleton, it is possible to instantiate 3^6^ (= 729) distinct grammars, with differing degrees of consistency in the ordering of sentence constituents. Reali and Christiansen implemented both biological and cultural evolution in their simulations: As with the evolution of better sequence learners, the initial weights of the network that best acquired a language in a given generation were slightly altered to produce the next generation of language learners—with the additional constraint that performance on the sequence learning task had to be maintained at the level reached at the end of the first part of the simulation (to capture the fact that humans are still superior sequence learners today). Cultural evolution of language was simulated by having the networks learn several different languages at each generation and then selecting the best learnt language as the basis for the next generation. The best learnt language was then varied slightly by changing the directions of a rule to produce a set of related “offspring” languages for each generation.

**Table 1:** **The grammar skeleton used by Reali and Christiansen (2009)**.

S → {NP VP}
NP → {N (PP)}
PP → {adp NP}
VP → {V (NP) (PP)}
NP → {N PossP}
PossP → {NP poss}

*S, sentence; NP, noun phrase; VP, verb phrase; PP, adpositional phrase; PossP, possessive phrase; N, noun; V, verb; adp, adposition; poss, possessive marker. Curly brackets indicate that the order of constituents can be as is, the reverse, or either way with equal probability (i.e., flexible word order). Parentheses indicate an optional constituent*.

Although the simulations started with language being completely flexible, and thus without any reliable word order constraints, after <100 generations of cultural evolution, the resulting language had adopted consistent word order constraints in all but one of the six rules. When comparing the networks from the first generation at which language was introduced and the final generation, Reali and Christiansen (2009) found no difference in linguistic performance. In contrast, when comparing network performance on the initial (all-flexible) language vs. the final language, a very large difference in learnability was observed. Together, these two analyses suggest that it was the cultural evolution of language, rather than biological evolution of better learners, that allowed language to become more easily learned and more structurally consistent across these simulations. More generally, the simulation results provide an existence proof that recursive structure can emerge in natural language by way of cultural evolution in the absence of language-specific constraints.

### Sequence learning and recursive consistency

An important remaining question is whether human learners are sensitive to the kind of sequence learning constraints revealed by Reali and Christiansen's (2009) simulated process of cultural evolution. A key result of these simulations was that the sequence learning constraints embedded in the SRNs tend to favor what we will refer to as *recursive consistency* (Christiansen and Devlin, 1997). Consider rewrite rules (2) and (3) from Table 1:

NP → {N (PP)}
PP → {adp NP}

Together, these two skeleton rules form a *recursive rule set* because each calls the other. Ignoring the flexible version of these two rules, we get the four possible recursive rule sets shown in Table 2. Using these rules sets we can generate the complex noun phrases seen in (3)–(6):

(3) [_NP_ *buildings* [_PP_ *from* [_NP_ *cities* [_PP_ *with* [_NP_ *smog*]]]]]
(4) [_NP_ [_PP_ [_NP_ [_PP_ [_NP_ *smog*] *with*] *cities*] *from*] *buildings*]
(5) [_NP_ *buildings* [_PP_ [_NP_ *cities* [_PP_ [_NP_ *smog*] *with*]] *from*]]
(6) [_NP_ [_PP_ *from* [_NP_ [_PP_ *with* [_NP_ *smog*]] *cities*]] *buildings*]

**Table 2:** **Recursive rule sets**.

**Right-branching**			**Left-branching**			**Mixed**			**Mixed**		
NP → N (PP)			NP → (PP) N			NP → N (PP)			NP → (PP) N		
PP → prep NP			PP → NP post			PP → NP post			PP → prep NP		

*NP, noun phrase; PP, adpositional phrase; prep, preposition; post, postposition; N, noun. Parentheses indicate an optional constituent*.

The first two rules sets from Table 2 generate recursively consistent structures that are either right-branching (as in 3) or left-branching (as in 4). The prepositions and postpositions, respectively, are always in close proximity to their noun complements, making it easier for a sequence learner to discover their relationship. In contrast, the final two rule sets generate recursively inconsistent structures, involving center-embeddings: all nouns are either stacked up before all the postpositions (5) or after all the prepositions (6). In both cases, the learner has to work out that *from* and *cities* together form a prepositional phrase, despite being separated from each other by another prepositional phrase involving *with* and *smog*. This process is further complicated by an increase in memory load caused by the intervening prepositional phrase. From a sequence learning perspective, it should therefore be easier to acquire the recursively consistent structure found in (3) and (4) compared with the recursively inconsistent structure in (5) and (6). Indeed, all the simulation runs in Reali and Christiansen (2009) resulted in languages in which both recursive rule sets were consistent.

Christiansen and Devlin (1997) had previously shown that SRNs perform better on recursively consistent structure (such as those in 3 and 4). However, if human language has adapted by way of cultural evolution to avoid recursive inconsistencies (such as 5 and 6), then we should expect people to be better at learning recursively consistent artificial languages than recursively inconsistent ones. Reeder (2004), following initial work by Christiansen (2000), tested this prediction by exposing participants to one of two artificial languages, generated by the artificial grammars shown in Table 3. Notice that the consistent grammar instantiates a left-branching grammar from the grammar skeleton used by Reali and Christiansen (2009), involving two recursively consistent rule sets (rules 2–3 and 5–6). The inconsistent grammar differs only in the direction of two rules (3 and 5), which are right-branching, whereas the other three rules are left-branching. The languages were instantiated using 10 spoken non-words to generate the sentences to which the participants were exposed. Participants in the two language conditions would see sequences of the exact same lexical items, only differing in their order of occurrence as dictated by the respective grammar (e.g., consistent: *jux vot hep vot meep nib* vs. inconsistent: *jux meep hep vot vot nib*). After training, the participants were presented with a new set of sequences, one by one, for which they were asked to judge whether or not these new items were generated by the same rules as the ones they saw previously. Half of the new items incorporated subtle violations of the sequence ordering (e.g., grammatical: *cav hep vot lum meep nib* vs. ungrammatical: *cav hep vot* rud *meep nib*, where *rud* is ungrammatical in this position).

**Table 3:** **The grammars used Christiansen (2000) and Reeder (2004)**.

**Consistent grammar**			**Inconsistent grammar**		
S → NP VP			S → NP VP		
NP → (PP) N			NP → (PP) N		
PP → NP post			PP → prep NP		
VP → (PP) (NP) V			VP → (PP) (NP) V		
NP → (PossP) N			NP → (PossP) N		
PossP → NP poss			PossP → poss NP		

*S, sentence; NP, noun phrase; VP, verb phrase; PP, adpositional phrase; PossP, possessive phrase; N, noun; V, verb; post, postposition; prep, preposition; poss, possessive marker. Parentheses indicate an optional constituent*.

The results of this artificial language learning experiment showed that the consistent language was learned significantly better (61.0% correct classification) than the inconsistent one (52.7%). It is important to note that because the consistent grammar was left-branching (and thus more like languages such as Japanese and Hindi), knowledge of English cannot explain the results. Indeed, if anything, the two right-branching rules in the inconsistent grammar bring that language closer to English^3^. To further demonstrate that the preferences for consistently recursive sequences is a domain-general bias, Reeder (2004) conducted a second experiment, in which the sequences were instantiated using black abstract shapes that cannot easily be verbalized. The results of the second study closely replicated those of the first, suggesting that there may be general sequence learning biases that favor recursively consistent structures, as predicted by Reali and Christiansen's (2009) evolutionary simulations.

The question remains, though, whether such sequence learning biases can drive cultural evolution of language in humans. That is, can sequence-learning constraints promote the emergence of language-like structure when amplified by processes of cultural evolution? To answer this question, Cornish et al. (under review) conducted an iterated sequence learning experiment, modeled on previous human iterated learning studies involving miniature language input (Kirby et al., 2008). Participants were asked to participate in a memory experiment, in which they were presented with 15 consonant strings. Each string was presented briefly on a computer screen after which the participants typed it in. After multiple repetitions of the 15 strings, the participants were asked to recall all of them. They were requested to continue recalling items until they had provided 15 unique strings. The recalled 15 strings were then recoded in terms of their specific letters to avoid trivial biases such as the location of letters on the computer keyboard and the presence of potential acronyms (e.g., *X* might be replaced throughout by *T, T* by *M*, etc.). The resulting set of 15 strings (which kept the same underlying structure as before recoding) was then provided as training strings for the next participant. A total of 10 participants were run within each “evolutionary” chain.

The initial set of strings used for the first participant in each chain was created so as to have minimal distributional structure (all consonant pairs, or bigrams, had a frequency of 1 or 2). Because recalling 15 arbitrary strings is close to impossible given normal memory constraints, it was expected that many of the recalled items would be strongly affected by sequence learning biases. The results showed that as these sequence biases became amplified across generations of learners, the sequences gained more and more distributional structure (as measured by the relative frequency of repeated two- and three-letter units). Importantly, the emerging system of sequences became more learnable. Initially, participants could only recall about 4 of the 15 strings correctly but by the final generation this had doubled, allowing participants to recall more than half the strings. Importantly, this increase in learnability did not evolve at the cost of string length: there was no decrease across generations. Instead, the sequences became easy to learn and recall because they formed a *system*, allowing subsequences to be reused productively. Using network analyses (see Baronchelli et al., 2013b, for a review), Cornish et al. demonstrated that the way in which this productivity was implemented strongly mirrored that observed for child-directed speech.

The results from Cornish et al. (under review) suggest that sequence learning constraints, as those explored in the simulations by Reali and Christiansen (2009) and demonstrated by Reeder (2004), can give rise to language-like distributional regularities that facilitate learning. This supports our hypothesis that sequential learning constraints, amplified by cultural transmission, could have shaped language into what we see today, including its limited use of embedded recursive structure. Next, we shall extend this approach to show how the same sequence learning constraints that we hypothesized to have shaped important aspects of the cultural evolution of recursive structures also can help explain specific patterns in the processing of complex recursive constructions.

### A usage-based account of complex recursive structure

So far, we have discussed converging evidence supporting the theory that language in important ways relies on evolutionarily prior neural mechanisms for sequence learning. But can a domain-general sequence learning device capture the ability of humans to process the kind of complex recursive structures that has been argued to require powerful grammar formalisms (e.g., Chomsky, 1956; Shieber, 1985; Stabler, 2009; Jäger and Rogers, 2012)? From our *usage-based* perspective, the answer does not necessarily require the postulation of recursive mechanisms as long as the proposed mechanisms can deal with the level of complex recursive structure that humans can actually process. In other words, what needs to be accounted for is the *empirical evidence* regarding human processing of complex recursive structures, and not *theoretical presuppositions* about recursion as a stipulated property of our language system.

**Figure 1:**
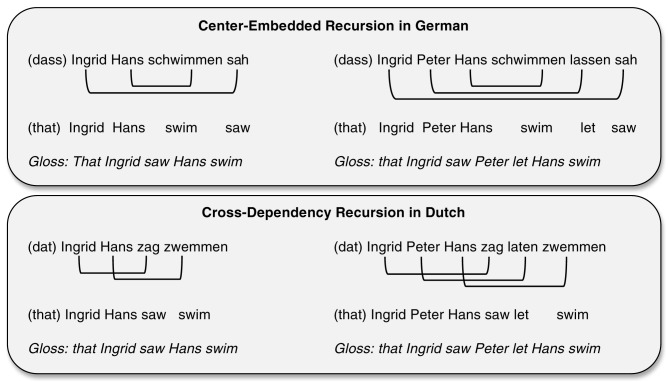
**Examples of complex recursive structures with one and two levels of embedding: Center-embeddings in German (top panel) and cross-dependencies in Dutch (bottom panel)**. The lines indicate noun-verb dependencies.

Christiansen and MacDonald (2009) conducted a set of computational simulations to determine whether a sequence-learning device such as the SRN would be able to capture human processing performance on complex recursive structures. Building on prior work by Christiansen and Chater (1999), they focused on the processing of sentences with center-embedded and cross-dependency structures. These two types of recursive constructions produce multiple overlapping non-adjacent dependencies, as illustrated in Figure 1, resulting in rapidly increasing processing difficulty as the number of embeddings grows. We have already discussed earlier how performance on center-embedded constructions breaks down at two levels of embedding (e.g., Wang, 1970; Hamilton and Deese, 1971; Blaubergs and Braine, 1974; Hakes et al., 1976). The processing of cross-dependencies, which exist in Swiss-German and Dutch, has received less attention, but the available data also point to a decline in performance with increased levels of embedding (Bach et al., 1986; Dickey and Vonk, 1997). Christiansen and MacDonald trained networks on sentences derived from one of the two grammars shown in Table 4. Both grammars contained a common set of recursive structures: right-branching recursive structure in the form of prepositional modifications of noun phrases, noun phrase conjunctions, subject relative clauses, and sentential complements; left-branching recursive structure in the form of prenominal possessives. The grammars furthermore had three additional verb argument structures (transitive, optionally transitive, and intransitive) and incorporated agreement between subject nouns and verbs. As illustrated by Table 4, the only difference between the two grammars was in the type of complex recursive structure they contained: center-embedding vs. cross-dependency.

**Table 4:** **The grammars used by Christiansen and MacDonald (2009)**.

**Rules common to both grammars**	
S → NP VP	
NP → N |NP PP |N *and* NP |N rel |PossP N	
PP → prep N (PP)	
rel_sub_ → *who* VP	
PossP → (PossP) N poss	
VP → V_i_ |V_t_ NP |V_o_ (NP) |V_c_ *that* S	
**Center-embedding grammar**	**Cross-dependency grammar**
rel_obj_ → *who* NP V_t|o_	S_cd_ → N_1_ N_2_ V_1(t|o)_ V_2(i)_
	S_cd_ → N_1_ N_2_ N V_1(t|o)_ V_2(t|o)_
	S_cd_ → N_1_ N_2_ N_3_ V_1(t|o)_ V_2(t|o)_ V_3(i)_
	S_cd_ → N_1_ N_2_ N_3_ N V_1(t|o)_ V_2(t|o)_ V _3(*t*|*o*)_

*S, sentence; NP, noun phrase; PP, prepositional phrase; PossP, possessive phrase; rel, relative clauses (subscripts, sub and obj, indicate subject/object relative clause); VP, verb phrase; N, noun; V, verb; prep, preposition; poss, possessive marker. For brevity, NP rules have been compressed into a single rule, using “|” to indicate exclusive options. The subscripts i, t, o, and c denote intransitive, transitive, optionally transitive, and clausal verbs, respectively. Subscript numbers indicate noun-verb dependency relations. Parentheses indicate an optional constituent*.

The grammars could generate a variety of sentences, with varying degree of syntactic complexity, from simple transitive sentences (such as 7) to more complex sentences involving different kinds of recursive structure (such as 8 and 9).

(7) John kisses Mary.
(8) Mary knows that John's boys' cats see mice.
(9) Mary who loves John thinks that men say that girls chase boys.

The generation of sentences was further restricted by probabilistic constraints on the complexity and depth of recursion. Following training on either grammar, the networks performed well on a variety of recursive sentence structures, demonstrating that the SRNs were able to acquire complex grammatical regularities (see also Christiansen, 1994)^4^. The networks acquired sophisticated abilities for generalizing across constituents in line with usage-based approaches to constituent structure (e.g., Beckner and Bybee, 2009; see also Christiansen and Chater, 1994). Differences between networks were observed, though, on their processing of the complex recursive structure permitted by the two grammars.

**Figure 2:**
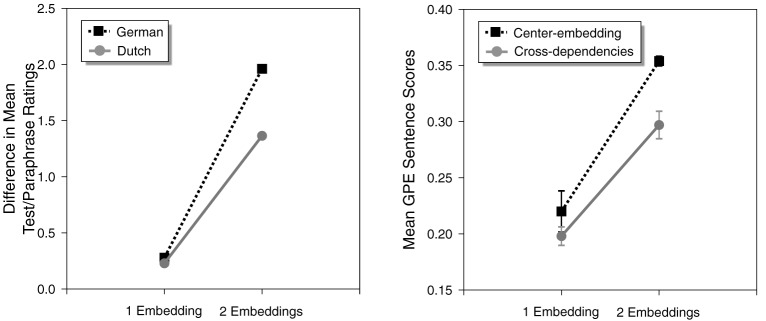
**Human performance (from Bach et al., 1986) on center-embedded constructions in German and cross-dependency constructions in Dutch with one or two levels of embedding (left)**. SRN performance on similar complex recursive structures (from Christiansen and MacDonald, 2009) **(right)**.

To model human data on the processing of center-embedding and cross-dependency structures, Christiansen and MacDonald (2009) relied on a study conducted by Bach et al. (1986) in which sentences with two center-embeddings in German were found to be significantly harder to process than comparable sentences with two cross-dependencies in Dutch. Bach et al. asked native Dutch speakers to rate the comprehensibility of Dutch sentences involving varying depths of recursive structure in the form of cross-dependency constructions and corresponding right-branching paraphrase sentences with similar meaning. Native speakers of German were tested using similar materials in German, where center-embedded constructions replaced the cross-dependency constructions. To remove potential effects of processing difficulty due to length, the ratings from the right-branching paraphrase sentences were subtracted from the complex recursive sentences. Figure 2 shows the results of the Bach et al. study on the left-hand side.

SRN performance was scored in terms of Grammatical Prediction Error (GPE; Christiansen and Chater, 1999), which measures the network's ability to make grammatically correct predictions for each upcoming word in a sentence, given prior context. The right-hand side of Figure 2 shows the mean sentence GPE scores, averaged across 10 novel sentences. Both humans and SRNs show similar qualitative patterns of processing difficulty (see also Christiansen and Chater, 1999). At a single level of embedding, there is no difference in processing difficulty. However, at two levels of embedding, cross-dependency structures (in Dutch) are processed more easily than comparable center-embedded structures (in German).

### Bounded recursive structure

Christiansen and MacDonald (2009) demonstrated that a sequence learner such as the SRN is able to mirror the differential human performance on center-embedded and cross-dependency recursive structures. Notably, the networks were able to capture human performance without the complex external memory devices (such as a stack of stacks; Joshi, 1990) or external memory constraints (Gibson, 1998) required by previous accounts. The SRNs ability to mimic human performance likely derives from a combination of *intrinsic* architectural constraints (Christiansen and Chater, 1999) and the distributional properties of the input to which it has been exposed (MacDonald and Christiansen, 2002; see also Christiansen and Chater, Forthcoming 2016). Christiansen and Chater (1999) analyzed the hidden unit representations of the SRN—its internal state—before and after training on recursive constructions and found that these networks have an architectural bias toward local dependencies, corresponding to those found in right-branching recursion. To process multiple instances of such recursive constructions, however, the SRN needs exposure to the relevant types of recursive structures. This exposure is particularly important when the network has to process center-embedded constructions because the network must overcome its architectural bias toward local dependencies. Thus, recursion is not a built-in property of the SRN; instead, the networks develop their human-like abilities for processing recursive constructions through repeated exposure to the relevant structures in the input.

As noted earlier, this usage-based approach to recursion differs from many previous processing accounts, in which unbounded recursion is implemented as part of the representation of linguistic knowledge (typically in the form of a rule-based grammar). Of course, this means that systems of the latter kind can process complex recursive constructions, such as center-embeddings, beyond human capabilities. Since Miller and Chomsky (1963), the solution to this mismatch has been to impose extrinsic memory limitations exclusively aimed at capturing human performance limitations on doubly center-embedded constructions (e.g., Kimball, 1973; Marcus, 1980; Church, 1982; Just and Carpenter, 1992; Stabler, 1994; Gibson and Thomas, 1996; Gibson, 1998; see Lewis et al., 2006, for a review).

To further investigate the nature of the SRN's intrinsic constraints on the processing of multiple center-embedded constructions, Christiansen and MacDonald (2009) explored a previous result from Christiansen and Chater (1999) showing that SRNs found ungrammatical versions of doubly center-embedded sentences with a missing verb more acceptable than their grammatical counterparts^5^ (for similar SRN results, see Engelmann and Vasishth, 2009). A previous offline rating study by Gibson and Thomas (1999) found that when the middle verb phrase (*was cleaning every week*) was removed from (10), the resulting ungrammatical sentence in (11) was rated no worse than the grammatical version in (10).

(10) The apartment that the maid who the service had sent over was cleaning every week was well decorated.
(11) ^*^The apartment that the maid who the service had sent over was well decorated.

However, when Christiansen and MacDonald tested the SRN on similar doubly center-embedded constructions, they obtained predictions for (11) to be rated better than (10). To test these predictions, they elicited *on-line* human ratings for the stimuli from the Gibson and Thomas study using a variation of the “stop making sense” sentence-judgment paradigm (Boland et al., 1990, 1995; Boland, 1997). Participants read a sentence, word-by-word, while at each step they decided whether the sentence was grammatical or not. Following the presentation of each sentence, participants rated it on a 7-point scale according to how good it seemed to them as a grammatical sentence of English (with 1 indicating that the sentence was “perfectly good English” and 7 indicating that it was “really bad English”). As predicted by the SRN, participants rated ungrammatical sentences such as (11) as better than their grammatical counterpart exemplified in (10).

The original stimuli from the Gibson and Thomas (1999) study had certain shortcomings that could have affected the outcome of the online rating experiment. Firstly, there were substantial length differences between the ungrammatical and grammatical versions of a given sentence. Secondly, the sentences incorporated semantic biases making it easier to line up a subject noun with its respective verb (e.g., *apartment–decorated, service–sent over* in 10). To control for these potential confounds, Christiansen and MacDonald (2009) replicated the experiment using semantically-neutral stimuli controlled for length (adapted from Stolz, 1967), as illustrated by (12) and (13).

(12) The chef who the waiter who the busboy offended appreciated admired the musicians.
(13) ^*^The chef who the waiter who the busboy offended frequently admired the musicians.

The second online rating experiment yielded the same results as the first, thus replicating the “missing verb” effect. These results have subsequently been confirmed by online ratings in French (Gimenes et al., 2009) and a combination of self-paced reading and eye-tracking experiments in English (Vasishth et al., 2010). However, evidence from German (Vasishth et al., 2010) and Dutch (Frank et al., in press) indicates that speakers of these languages do not show the missing verb effect but instead find the grammatical versions easier to process. Because verb-final constructions are common in German and Dutch, requiring the listener to track dependency relations over a relatively long distance, substantial prior experience with these constructions likely has resulted in language-specific processing improvements (see also Engelmann and Vasishth, 2009; Frank et al., in press, for similar perspectives). Nonetheless, in some cases the missing verb effect may appear even in German, under conditions of high processing load (Trotzke et al., 2013). Together, the results from the SRN simulations and human experimentation support our hypothesis that the processing of center-embedded structures are best explained from a usage-based perspective that emphasizes processing experience with the specific statistical properties of individual languages. Importantly, as we shall see next, such linguistic experience interacts with sequence learning constraints.

### Sequence learning limitations mirror constraints on complex recursive structure

Previous studies have suggested that the processing of singly embedded relative clauses are determined by linguistic experience, mediated by sequence learning skills (e.g., Wells et al., 2009; Misyak et al., 2010; see Christiansen and Chater, Forthcoming 2016, for discussion). Can our limited ability to process multiple complex recursive embeddings similarly be shown to reflect constraints on sequence learning? The embedding of multiple complex recursive structures—whether in the form of center-embeddings or cross-dependencies—results in several pairs of overlapping non-adjacent dependencies (as illustrated by Figure 1). Importantly, the SRN simulation results reported above suggest that a sequence learner might also be able to deal with the increased difficulty associated with multiple, overlapping non-adjacent dependencies.

Dealing appropriately with multiple non-adjacent dependencies may be one of the key defining characteristics of human language. Indeed, when a group of generativists and cognitive linguists recently met to determine what is special about human language (Tallerman et al., 2009), one of the few things they could agree about was that long-distance dependencies constitute one of the hallmarks of human language, and not recursion (contra Hauser et al., 2002). de Vries et al. (2012) used a variation of the AGL-SRT task (Misyak et al., 2010) to determine whether the limitations on processing of multiple non-adjacent dependencies might depend on general constraints on human sequence learning, instead of being unique to language. This task incorporates the structured, probabilistic input of artificial grammar learning (AGL; e.g., Reber, 1967) within a modified two-choice serial reaction-time (SRT; Nissen and Bullemer, 1987) layout. In the de Vries et al. study, participants used the computer mouse to select one of two written words (a target and a foil) presented on the screen as quickly as possible, given auditory input. Stimuli consisted of sequences with two or three non-adjacent dependencies, ordered either using center-embeddings or cross-dependencies. The dependencies were instantiated using a set of dependency pairs that were matched for vowel sounds: *ba-la, yo-no, mi-di*, and *wu-tu*. Examples of each of the four types of stimuli are presented in (14–17), where the subscript numbering indicates dependency relationships.

(14) ba_1_ wu_2_ tu_2_ la_1_
(15) ba_1_ wu_2_ la_1_ tu_2_
(16) ba_1_ wu_2_ yo_3_ no_3_ tu_2_ la_1_
(17) ba_1_ wu_2_ yo_3_ la_1_ tu_2_ no_3_

Thus, (14) and (16) implement center-embedded recursive structure and (15) and (17) involve cross-dependencies. Participants would only be exposed to one of the four types of stimuli. To determine the potential effect of linguistic experience on the processing of complex recursive sequence structure, study participants were either native speakers of German (which has center-embedding but not cross-dependencies) or Dutch (which has cross-dependencies). Participants were only exposed to one kind of stimulus, e.g., doubly center-embedded sequences as in (16) in a fully crossed design (length × embedding × native language).

de Vries et al. (2012) first evaluated learning by administering a block of ungrammatical sequences in which the learned dependencies were violated. As expected, the ungrammatical block produced a similar pattern of response slow-down for both for both center-embedded and cross-dependency items involving two non-adjacent dependencies (similar to what Bach et al., 1986, Bach et al., found in the natural language case). However, an analog of the missing verb effect was observed for the center-embedded sequences with three non-adjacencies but not for the comparable cross-dependency items. Indeed, an incorrect middle element in the center-embedded sequences (e.g., where *tu* is replaced by *la* in 16) did not elicit any slow-down at all, indicating that participants were not sensitive to violations at this position.

Sequence learning was further assessed using a prediction task at the end of the experiment (after a recovery block of grammatical sequences). In this task, participants would hear a beep replacing one of the elements in the second half of the sequence and were asked to simply click on the written word that they thought had been replaced. Participants exposed to the sequences incorporating two dependencies, performed reasonably well on this task, with no difference between center-embedded and cross-dependency stimuli. However, as for the response times, a missing verb effect was observed for the center-embedded sequences with three non-adjacencies. When the middle dependent element was replaced by a beep in center-embedded sequences (e.g., *ba*_1_ *wu*_2_ *yo*_3_ *no*_3_<*beep*> *la*_1_), participants were more likely to click on the foil (e.g., la) than the target (tu). This was not observed for the corresponding cross-dependency stimuli, once more mirroring the Bach et al. (1986) psycholinguistic results that multiple cross-dependencies are easier to process than multiple center-embeddings.

Contrary to psycholinguistic studies of German (Vasishth et al., 2010) and Dutch (Frank et al., in press), de Vries et al. (2012) found an analog of the missing verb effect in speakers of both languages. Because the sequence-learning task involved non-sense syllables, rather than real words, it may not have tapped into the statistical regularities that play a key role in real-life language processing^6^. Instead, the results reveal fundamental limitations on the learning and processing of complex recursively structured sequences. However, these limitations may be mitigated to some degree, given sufficient exposure to the “right” patterns of linguistic structure—including statistical regularities involving morphological and semantic cues—and thus lessening sequence processing constraints that would otherwise result in the missing verb effect for doubly center-embedded constructions. Whereas the statistics of German and Dutch appear to support such amelioration of language processing, the statistical make-up of linguistic patterning in English and French apparently does not. This is consistent with the findings of Frank et al. (in press), demonstrating that native Dutch and German speakers show a missing verb effect when processing English (as a second language), even though they do not show this effect in their native language (except under extreme processing load, Trotzke et al., 2013). Together, this pattern of results suggests that the constraints on human processing of multiple long-distance dependencies in recursive constructions stem from limitations on sequence learning interacting with linguistic experience.

### Summary

In this extended case study, we argued that our ability to process of recursive structure does not rely on recursion as a property of the grammar, but instead emerges gradually by piggybacking on top of domain-general sequence learning abilities. Evidence from genetics, comparative work on non-human primates, and cognitive neuroscience suggests that humans have evolved complex sequence learning skills, which were subsequently pressed into service to accommodate language. Constraints on sequence learning therefore have played an important role in shaping the cultural evolution of linguistic structure, including our limited abilities for processing recursive structure. We have shown how this perspective can account for the degree to which humans are able to process complex recursive structure in the form of center-embeddings and cross-dependencies. Processing limitations on recursive structure derive from constraints on sequence learning, modulated by our individual native language experience.

We have taken the first steps toward an evolutionarily-informed usage-based account of recursion, where our recursive abilities are acquired piecemeal, construction by construction, in line with developmental evidence. This perspective highlights the key role of language experience in explaining cross-linguistic similarities and dissimilarities in the ability to process different types of recursive structure. And although, we have focused on the important role of sequence learning in explaining the limitations of human recursive abilities, we want to stress that language processing, of course, includes other domain-general factors. Whereas distributional information clearly provides important input to language acquisition and processing, it is not sufficient, but must be complemented by numerous other sources of information, from phonological and prosodic cues to semantic and discourse information (e.g., Christiansen and Chater, 2008, Forthcoming 2016). Thus, our account is far from complete but it does offer the promise of a usage-based perspective of recursion based on evolutionary considerations.

## Language without a language faculty

In this paper, we have argued that there are theoretical reasons to suppose that special-purpose biological machinery for language can be ruled out on evolutionary grounds. A possible counter-move adopted by the minimalist approach to language is to suggest that the faculty of language is very minimal and only consists of recursion (e.g., Hauser et al., 2002; Chomsky, 2010). However, we have shown that capturing human performance on recursive constructions does not require an innate mechanism for recursion. Instead, we have suggested that the variation in processing of recursive structures as can be observed across individuals, development and languages is best explained by domain-general abilities for sequence learning and processing interacting with linguistic experience. But, if this is right, it becomes crucial to provide explanations for the puzzling aspects of language that were previously used to support the case for a rich innate language faculty: (1) the poverty of the stimulus, (2) the eccentricity of language, (3) language universals, (4) the source of linguistic regularities, and (5) the uniqueness of human language. In the remainder of the paper, we therefore address each of these five challenges, in turn, suggesting how they may be accounted for without recourse to anything more than domain-general constraints.

### The poverty of the stimulus and the possibility of language acquisition

One traditional motivation for postulating an innate language faculty is the assertion that there is insufficient information in the child's linguistic environment for reliable language acquisition to be possible (Chomsky, 1980). If the language faculty has been pared back to consist only of a putative mechanism for recursion, then this motivation no longer applies—the complex patterns in language which have been thought to pose challenges of learnability concern highly specific properties of language (e.g., concerning binding constraints), which are not resolved merely by supplying the learner with a mechanism for recursion.

But recent work provides a positive account of how the child can acquire language, in the absence of an innate language faculty, whether minimal or not. One line of research has shown, using computational results from language corpora and mathematical analysis, that learning methods are much more powerful than had previously been assumed (e.g., Manning and Schütze, 1999; Klein and Manning, 2004; Chater and Vitányi, 2007; Hsu et al., 2011, 2013; Chater et al., 2015). But more importantly, viewing language as a culturally evolving system, shaped by the selectional pressures from language learners, explains why language and languages learners fit together so closely. In short, the remarkable phenomenon of language acquisition from a noisy and partial linguistic input arises from a close fit between the structure of language and the structure of the language learner. However, the origin of this fit is not that the learner has somehow acquired a special-purpose language faculty embodying universal properties of human languages, but, instead, because language has been subject to powerful pressures of cultural evolution to match, as well as possible, the learning and processing mechanism of its speakers (e.g., as suggested by Reali and Christiansen's, 2009, simulations). In short, the brain is not shaped for language; language is shaped by the brain (Christiansen and Chater, 2008).

Language acquisition can overcome the challenges of the poverty of the stimulus without recourse to an innate language faculty, in light both of new results on learnability, and the insight that language has been shaped through processes of cultural evolution to be as learnable as possible.

### The eccentricity of language

Fodor (1983) argue that the generalizations found in language are so different from those evident in other cognitive domains, that they can only be subserved by highly specialized cognitive mechanisms. But the cultural evolutionary perspective that we have outlined here suggests, instead, that the generalizations observed in language are not so eccentric after all: they arise, instead, from a wide variety of cognitive, cultural, and communicative constraints (e.g., as exemplified by our extended case study of recursion). The interplay of these constraints, and the contingencies of many thousands of years of cultural evolution, is likely to have resulted in the apparently baffling complexity of natural languages.

### Universal properties of language

Another popular motivation for proposing an innate language faculty is to explain putatively universal properties across all human languages. Such universals can be explained as consequences of the innate language faculty—and variation between languages has often been viewed as relatively superficial, and perhaps as being determined by the flipping of a rather small number of discrete “switches,” which differentiate English, Hopi and Japanese (e.g., Lightfoot, 1991; Baker, 2001; Yang, 2002).

By contrast, we see “universals” as products of the interaction between constraints deriving from the way our thought processes work, from perceptuo-motor factors, from cognitive limitations on learning and processing, and from pragmatic sources. This view implies that most universals are unlikely to be found across all languages; rather, “universals” are more akin to statistical trends tied to patterns of language use. Consequently, specific universals fall on a continuum, ranging from being attested to only in some languages to being found across most languages. An example of the former is the class of implicational universals, such as that verb-final languages tend to have postpositions (Dryer, 1992), whereas the presence of nouns and verbs (minimally as typological prototypes; Croft, 2001) in most, though perhaps not all (Evans and Levinson, 2009), languages is an example of the latter.

Individual languages, on our account, are seen as evolving under the pressures from multiple constraints deriving from the brain, as well as cultural-historical factors (including language contact and sociolinguistic influences), resulting over time in the breathtaking linguistic diversity that characterize the about 6–8000 currently existing languages (see also Dediu et al., 2013). Languages variously employ tones, clicks, or manual signs to signal differences in meaning; some languages appear to lack the noun-verb distinction (e.g., Straits Salish), whereas others have a proliferation of fine-grained syntactic categories (e.g., Tzeltal); and some languages do without morphology (e.g., Mandarin), while others pack a whole sentence into a single word (e.g., Cayuga). Cross-linguistically recurring patterns do emerge due to similarity in constraints and culture/history, but such patterns should be expected to be probabilistic tendencies, not the rigid properties of a universal grammar (Christiansen and Chater, 2008). From this perspective it seems unlikely that the world's languages will fit within a single parameterized framework (e.g., Baker, 2001), and more likely that languages will provide a diverse, and somewhat unruly, set of solutions to a hugely complex problem of multiple constraint satisfaction, as appears consistent with research on language typology (Comrie, 1989; Evans and Levinson, 2009; Evans, 2013). Thus, we construe recurring patterns of language along the lines of Wittgenstein's (1953) notion of “family resemblance”: although there may be similarities between pairs of individual languages, there is no single set of features common to all.

### Where do linguistic regularities come from?

Even if the traditional conception of language universals is too strict, the challenge remains: in the absence of a language faculty, how can we explain why language is orderly at all? How is it that the processing of myriads of different constructions have not created a chaotic mass of conflicting conventions, but a highly, if partially, structured system linking form and meaning?

The spontaneous creation of tracks in a forest provides an interesting analogy (Christiansen and Chater, in press). Each time an animal navigates through the forest, it is concerned only with reaching its immediate destination as easily as possible. But the cumulative effect of such navigating episodes, in breaking down vegetation and gradually creating a network of paths, is by no means chaotic. Indeed, over time, we may expect the pattern of tracks to become increasingly ordered: kinks will be become straightened; paths between ecological salient locations (e.g., sources of food, shelter or water) will become more strongly established; and so on. We might similarly suspect that language will become increasingly ordered over long periods of cultural evolution.

We should anticipate that such order should emerge because the cognitive system does not merely learn lists of lexical items and constructions by rote; it generalizes from past cases to new cases. To the extent that the language is a disordered morass of competing and inconsistent regularities, it will be difficult to process and difficult to learn. Thus, the cultural evolution of language, both within individuals and across generations of learners, will impose a strong selection pressure on individual lexical items and constructions to align with each other. Just as stable and orderly forest tracks emerge from the initially arbitrary wanderings of the forest fauna, so an orderly language may emerge from what may, perhaps, have been the rather limited, arbitrary and inconsistent communicative system of early “proto-language.” In particular, for example, the need to convey an unlimited number of messages will lead to a drive to recombine linguistic elements is systematic ways, yielding increasingly “compositional” semantics, in which the meaning of a message is associated with the meaning of its parts, and the way in which they are composed together (e.g., Kirby, 1999, 2000).

### Uniquely human?

There appears to be a qualitative difference between communicative systems employed by non-human animals, and human natural language: one possible explanation is that humans, alone, possess an innate faculty for language. But human “exceptionalism” is evident in many domains, not just in language; and, we suggest, there is good reason to suppose that what makes humans special concerns aspect of our cognitive and social behavior, which evolved prior to the emergence of language, but made possible the collective construction of natural languages through long processes of cultural evolution.

A wide range of possible cognitive precursors for language have been proposed. For example, human sequence processing abilities for complex patterns, described above, appear significantly to outstrip processing abilities of non-human animals (e.g., Conway and Christiansen, 2001). Human articulatory machinery may be better suited to spoken language than that of other apes (e.g., Lieberman, 1968). And the human abilities to understand the minds of others (e.g., Call and Tomasello, 2008) and to share attention (e.g., Knoblich et al., 2011) and to engage in joint actions (e.g., Bratman, 2014), may all be important precursors for language.

Note, though, that from the present perspective, language is continuous with other aspects of culture—and almost all aspects of human culture, from music and art to religious ritual and belief, moral norms, ideologies, financial institutions, organizations, and political structures are uniquely human. It seems likely that such complex cultural forms arise through long periods of cultural innovation and diffusion, and that the nature of such propagation depends will depend on a multitude of historical, sociological, and, most likely, a host of cognitive factors (e.g., Tomasello, 2009; Richerson and Christiansen, 2013). Moreover, we should expect that different aspects of cultural evolution, including the evolution of language, will be highly interdependent. In the light of these considerations, once the presupposition that language is *sui generis* and rooted in a genetically-specified language faculty is abandoned, there seems little reason to suppose that there will be a clear-cut answer concerning the key cognitive precursors for human language, any more than we should expect to be able to enumerate the precursors of cookery, dancing, or agriculture.

## Language as culture, not biology

Prior to the seismic upheavals created by the inception of generative grammar, language was generally viewed as a paradigmatic, and indeed especially central, element of human culture. But the meta-theory of the generative approach was taken to suggest a very different viewpoint: that language is primarily a biological, rather than a cultural, phenomenon: the knowledge of the language was seen not as embedded in a culture of speakers and hearers, but primarily in a genetically-specified language faculty.

We suggest that, in light of the lack of a plausible evolutionary origin for the language faculty, and a re-evaluation of the evidence for even the most minimal element of such a faculty, the mechanism of recursion, it is time to return to viewing language as a cultural, and not a biological, phenomenon. Nonetheless, we stress that, like other aspects of culture, language will have been shaped by human processing and learning biases. Thus, understanding the structure, acquisition, processing, and cultural evolution of natural language requires unpicking how language has been shaped by the biological and cognitive properties of the human brain.

### Conflict of interest statement

The authors declare that the research was conducted in the absence of any commercial or financial relationships that could be construed as a potential conflict of interest.

## Footnotes

^1^Although we do not discuss sign languages explicitly in this article, we believe that they are subject to the same arguments as we here present for spoken language. Thus, our arguments are intended to apply to language in general, independently of the modality within which it is expressed (see Christiansen and Chater, Forthcoming 2016, in press, for further discussion).

^2^Some studies purportedly indicate that the mechanisms involved in syntactic language processing are not the same as those involved in most sequence learning tasks (e.g., Penã et al., 2002; Musso et al., 2003; Friederici et al., 2006). However, the methods and arguments used in these studies have subsequently been challenged (de Vries et al., 2008; Marcus et al., 2003, and Onnis et al., 2005, respectively), thereby undermining their negative conclusions. Overall, the preponderance of the evidence suggests that sequence-learning tasks tap into the mechanisms involved in language acquisition and processing (see Petersson et al., 2012, for discussion).

^3^We further note that the SRN simulations by Christiansen and Devlin (1997) showed a similar pattern, suggesting that a general linguistic capacity is not required to explain these results. Rather, the results would appear to arise from the distributional patterns inherent to the two different artificial grammars.

^4^All simulations were replicated multiple times (including with variations in network architecture and corpus composition), yielding qualitatively similar results.

^5^Importantly, Christiansen and Chater (1999) demonstrated that this prediction is primarily due to intrinsic architectural limitations on the processing on doubly center-embedded material rather than insufficient experience with these constructions. Moreover, they further showed that the intrinsic constraints on center-embedding are independent of the size of the hidden unit layer.

^6^de Vries et al. (2012) did observe a nontrivial effect of language exposure: German speakers were faster at responding to center-embedded sequences with two non-adjacencies than to the corresponding cross-dependency stimuli. No such difference was found for the Germans learning the sequences with three nonadjacent dependencies, nor did the Dutch participants show any response-time differences across any of the sequence types. Given that center-embedded constructions with two dependencies are much more frequent than with three dependencies (see Karlsson, 2007, for a review), this pattern of differences may reflect the German participants' prior linguistic experience with center-embedded, verb-final constructions.
